# Supplementary material for: MicroRNAs as potential therapeutics to enhance chemosensitivity in advanced prostate cancer
Source: Sci Rep. 2018 May 18;8:7820. doi: 10.1038/s41598-018-26050-y (PMC5959911; doi:10.1038/s41598-018-26050-y)
Supplement: Supplementary file 1 — Supplementary Information [file 41598_2018_26050_MOESM1_ESM.pdf]

Supplementary Information for:

MicroRNAs as potential therapeutics to enhance chemosensitivity in advanced prostate cancer

Authors

Hui-Ming Lin[1,2], Iva Nikolic[1,2,3], Jessica Yang[1], Lesley Castillo[1], Niantao Deng[1,2], Chia-Ling Chan[1], Nicole K. Yeung[1], Eoin Dodson[1], Benjamin Elsworth[1], Calan Spielman[1], Brian Y. Lee[1,4], Zoe Boyer[1], Kaylene J. Simpson[3,5], Roger J. Daly[6,7], Lisa G. Horvath[1,8,9]*, Alexander Swarbrick[1,2]*

[1]Cancer Division, The Kinghorn Cancer Centre, Garvan Institute of Medical Research, Darlinghurst, New South Wales 2010, Australia
[2]St Vincent's Clinical School, UNSW Sydney, New South Wales 2010, Australia
[3]Victorian Centre for Functional Genomics, Peter MacCallum Cancer Centre, Melbourne, Victoria 3000, Australia
[4]current address: Systems Oncology, Cancer Research UK Manchester Institute, The University of Manchester, Manchester M20 4QL, United Kingdom
[5]The Sir Peter MacCallum Department of Oncology, University of Melbourne, Parkville, Victoria 3000, Australia
[6]Signalling Network Laboratory, Department of Biochemistry and Molecular Biology, Monash University, Clayton, Victoria 3800, Australia;
[7]Cancer Program, Biomedicine Discovery Institute, Monash University, Clayton, Victoria 3800, Australia
[8]Medical Oncology, Chris O'Brien Lifehouse, Camperdown, New South Wales 2050, Australia
[9]Sydney Medical School, University of Sydney, Camperdown, New South Wales 2050, Australia

*LGH and AS are co-corresponding authors
Corresponding authors:
Associate Professor Alexander Swarbrick; Tumour Progression Laboratory, Cancer Division, The Kinghorn Cancer Centre, Garvan Institute of Medical Research, 384 Victoria Street, Darlinghurst, New South Wales 2010, Australia. Phone: +61-2-93555780; email: a.swarbrick@garvan.org.au
Professor Lisa G. Horvath; Department of Medical Oncology, Chris O'Brien Lifehouse, PO BOX M33, Missenden Road, Camperdown, New South Wales 2050, Australia. Phone: +61-2-85140142; email: lisa.horvath@lh.org.au

Figure S1.
Identification of microRNA inhibitors with taxane sensitisation effects in PC3 and DU145 cell lines in the functional screen testing 1280 microRNA inhibitors in combination with docetaxel or cabazitaxel treatment: (A) Number of hits at different thresholds of taxane sensitisation; (B) Viability plots showing that none of the microRNA inhibitors have the same degree of taxane sensitisation as those microRNA mimics with the strongest effect when the same threshold was used.

A

% viability, vehicle*	% viability, taxane*	Number of hits from screen with microRNA inhibitors			
		PC3		DU145	
		docetaxel	cabazitaxel	docetaxel	cabazitaxel
>80%	<80%	71	37	29	22
	<70%	14	15	3	2
	<60%	0	5	0	0
	<50%	0	0	0	0

* Viability is relative to that of non-targeting control with respective treatment

B

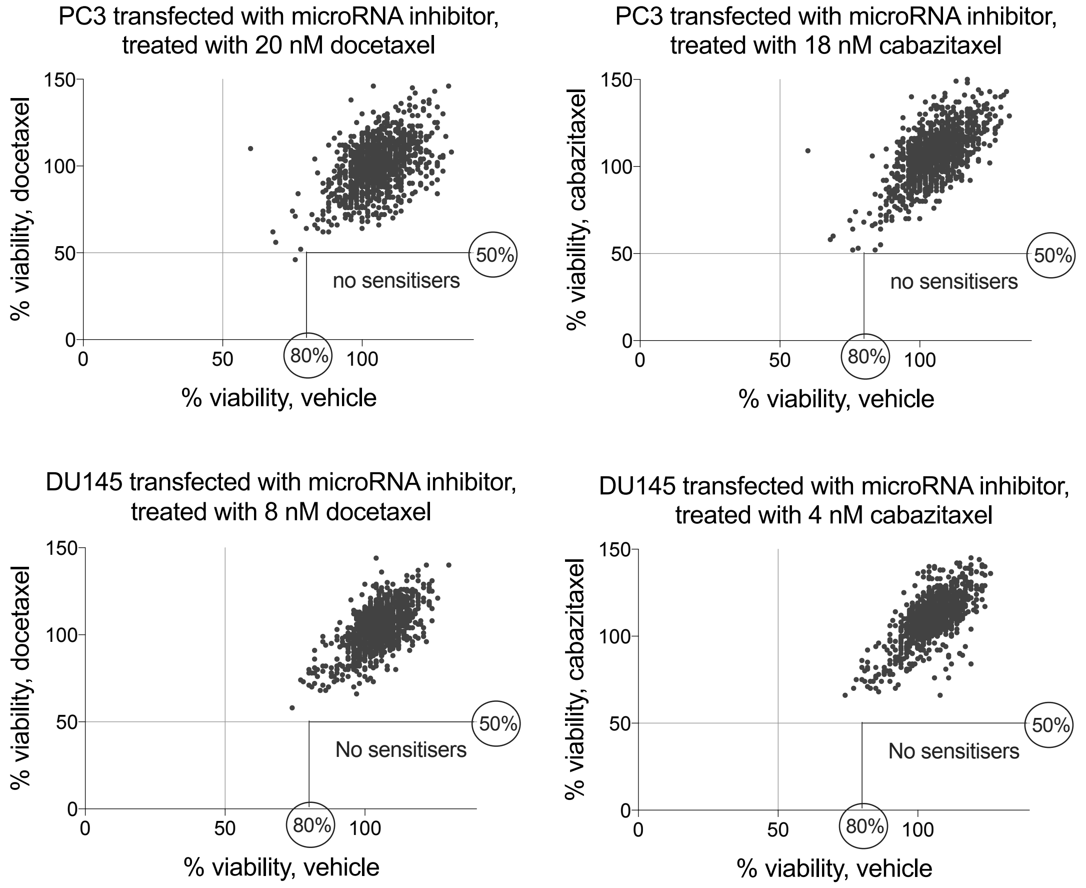

Figure S2.
Growth curves, measured by IncuCyte Zoom, of PC3 cells transfected with microRNA mimics or non-targeting control from replicate experiments (see Figure 2b in main article). Each curve is from a single technical replicate per experiment (1 well of a 6 well multiwell plate).

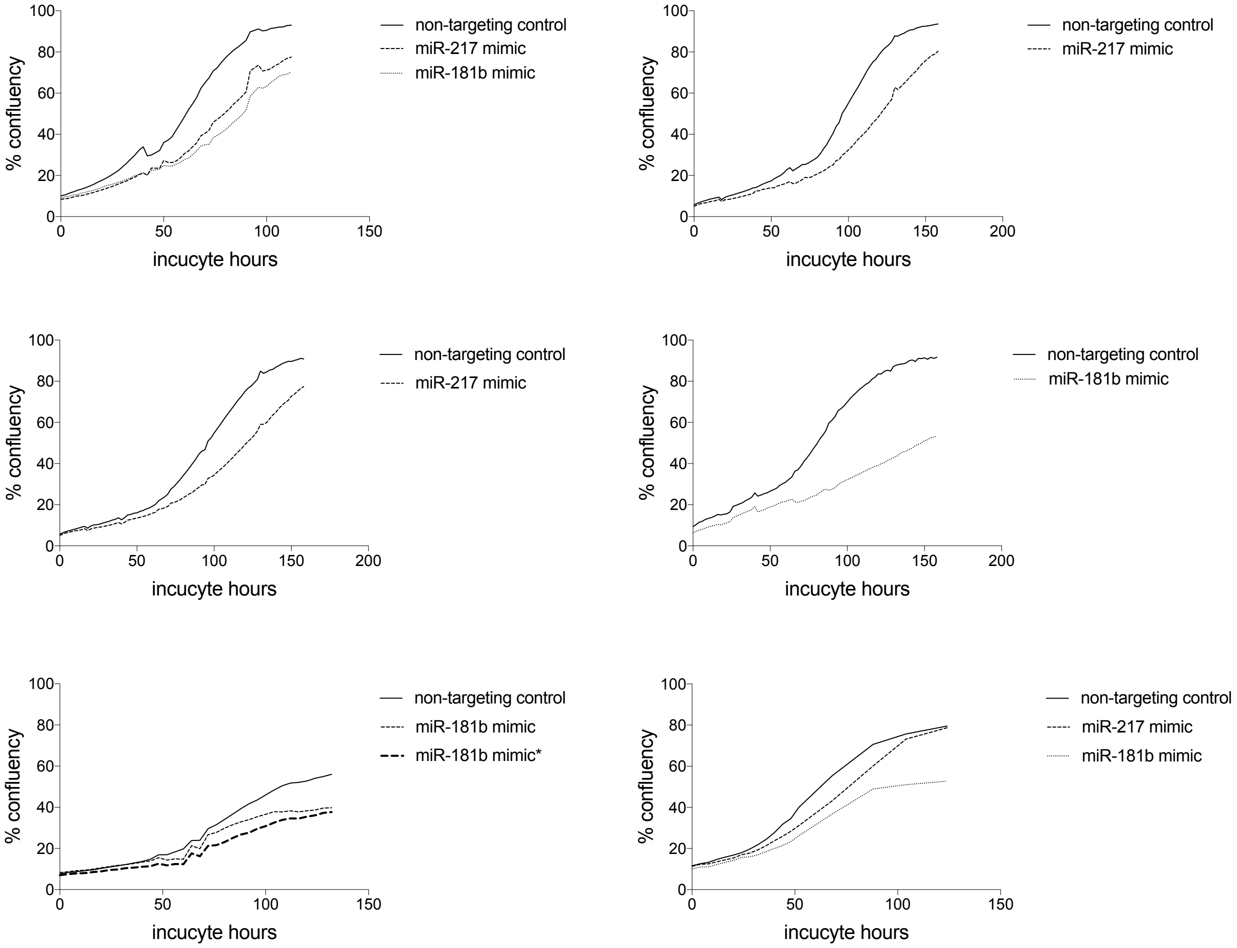

Figure S3.

Mir-100-3p and miR-153 mimics are toxic but do not increase the sensitivity of PC3 and DU145 to cabazitaxel respectively: (A) Cabazitaxel IC50 or maximum response for cells transfected with the mimics were not significantly different from those transfected with non-targeting control (P>0.05; mean ± standard deviation of 3 experiments with 6 replicate samples each; cell viability assessed with alamarBlue Cell Viability assay in 96-well microplates); (B) Viability of cells transfected with mimics were lower compared to those transfected with non-targeting control, without cabazitaxel treatment (viability represented by fluorescence measurements from alamarBlue Cell Viability assay; bars are mean ± standard deviation).

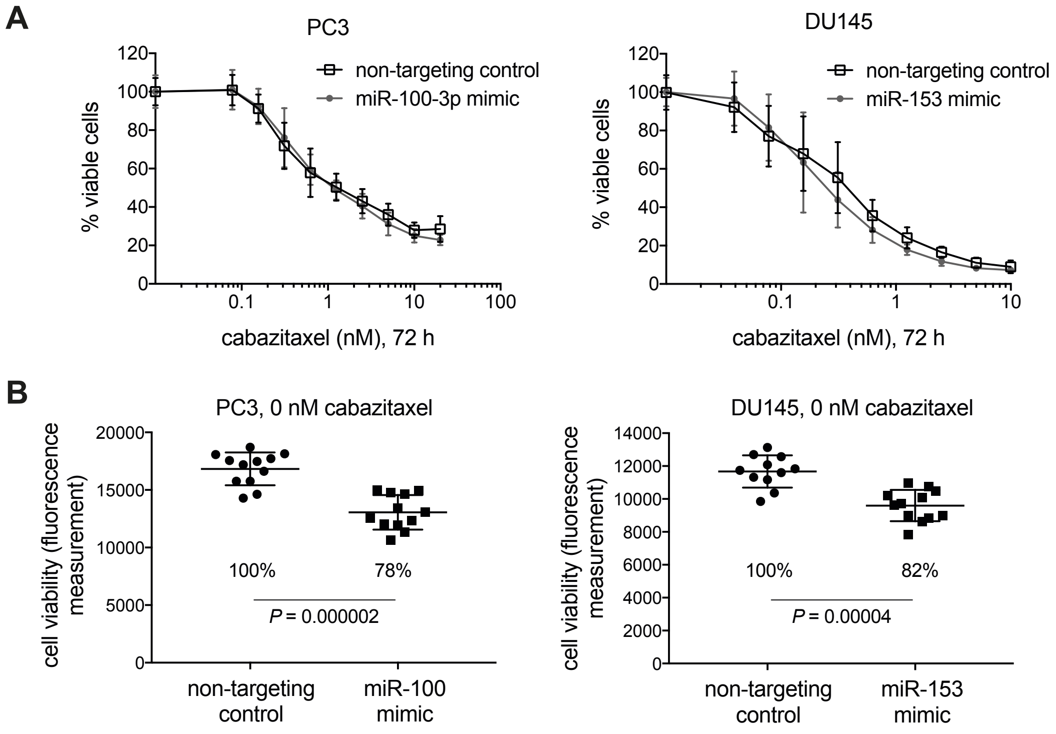

Figure S4.

MiR-217 and miR-181b mimics do not increase taxane sensitivity or decrease viability of DU145 cells: (A) Taxane IC50 or maximum effect for cells transfected with the mimics were not significantly different from those transfected with non-targeting control (P>0.05; mean ± standard deviation of 4 replicate samples, cell viability assessed with CellTiter-Glo Luminescent Cell Viability assay in 96-well microplates); (B) Percentage of apoptotic cells was not enhanced by transfection with mimics (quantitated by flow cytometry with annexin-V-FITC and propidium iodide labelling (P>0.05; mean ± standard deviation of 3 experiments).

A

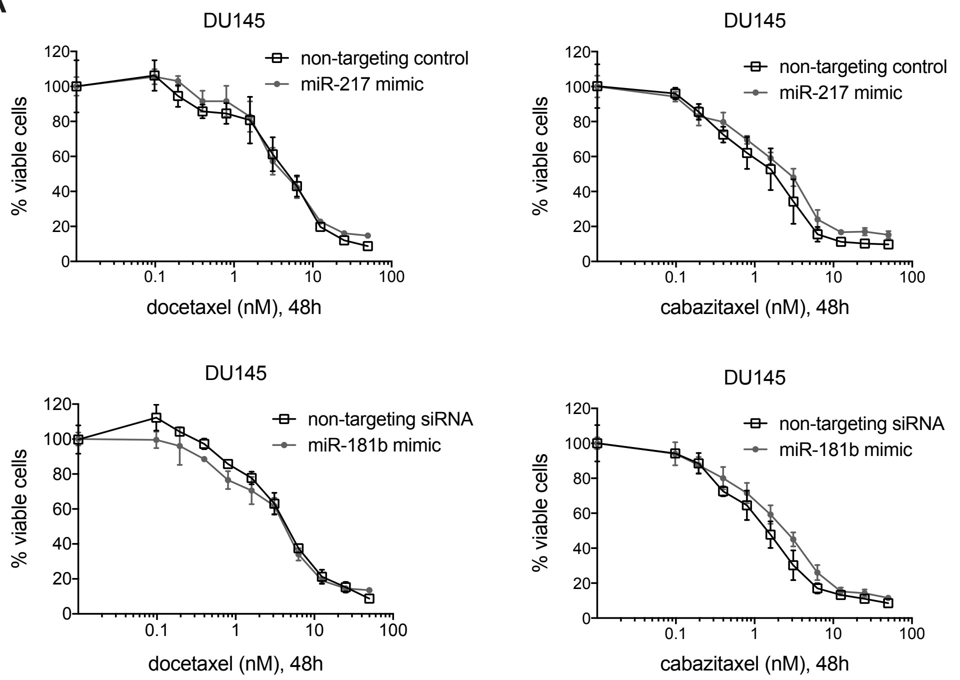

B

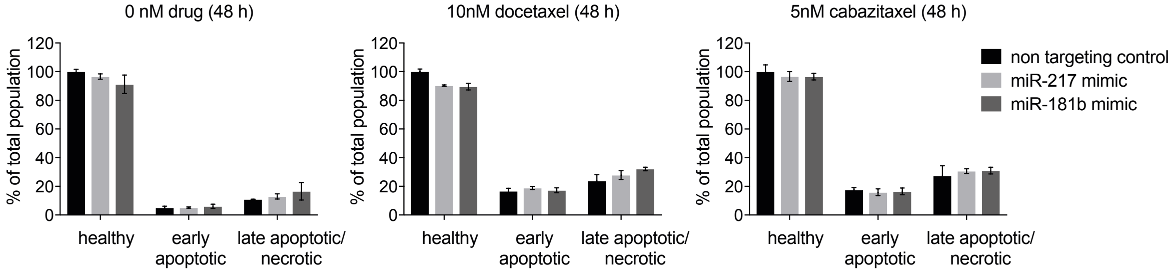

Figure S5.
MiR-181b-5p expression in human prostate tumours and non-malignant prostate tissue (line represents median). The data was generated by Taylor *et al* using microRNA microarray[1] and the normalised data was obtained from cBioPortal. MiR-217 levels were not in the normalised data for any of the tissues even though its probe was present on the microarray.

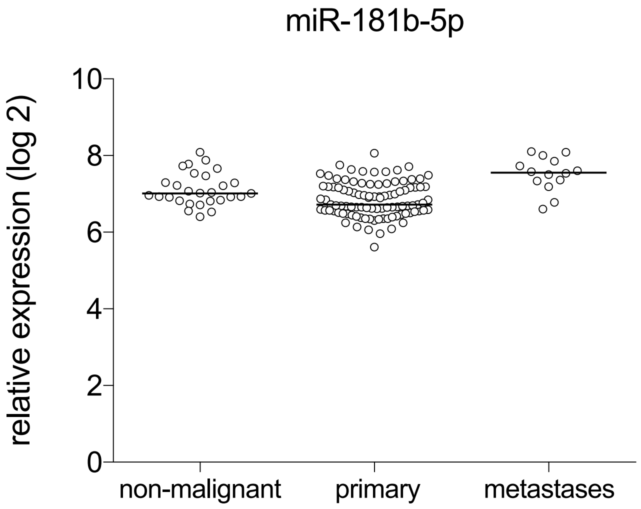

number of samples: *28* *99* *14*

[1]Taylor BS, Schultz N, Hieronymus H, Gopalan A, Xiao Y, Carver B S, Arora V K, Kaushik P, Cerami E, Reva B, Antipin Y, Mitsiades N, Landers T, Dolgalev I, Major JE, Wilson M, Socci N D, Lash A E, Heguy A, Eastham JA, Scher HI, Reuter VE, Scardino PT, Sander C, Sawyers CL, Gerald WL. Integrative genomic profiling of human prostate cancer. *Cancer Cell* 2010; 18(1): 11-22

Table S1.

Genes selected for siRNA knockdown. The transcripts of these genes were significantly downregulated in PC3 cells transfected with miR-217 or miR-181b-5p mimic compared to non-targeting control according to RNA-sequencing. (Abbreviations: RPKM, reads per kilobase per million mapped reads; FDR, false discovery rate)

Reason for selection	Gene symbol	Description	Non-targeting control / miR-217 mimic			Non-targeting control / miR-181b-5p mimic		
			RPKM fold change	FDR adjusted P-value	Signi-ficance rank	RPKM fold change	FDR adjusted P-value	Signi-ficance rank
cell cycle function	PLK1	polo like kinase 1	-	-	-	1.6	$2.6 x10^{-12}$	1002
	FBXO5	F-box protein 5	1.4	$7.2x10^{-8}$	793	2.8	$2.1 x10^{-74}$	57
	TUBA1A	tubulin alpha 1a	2.1	$7.9x10^{-49}$	91	3.4	$6.1 x10^{-126}$	11
	ANLN	anillin actin binding protein	2.7	$1.8x10^{-50}$	86	1.7	$3.0 x10^{-16}$	827
	YWHAZ	tyrosine 3-monooxygenase/tryptophan 5-monooxygenase activation protein zeta	2.2	$1.2x10^{-75}$	32	1.6	$4.9 x10^{-29}$	393
	MCM3	minichromosome maintenance complex component 3	2.2	$2.4x10^{-60}$	59	1.5	$1.2 x10^{-17}$	779
	HJURP	Holliday junction recognition protein	1.2	$1.1x10^{-05}$	986	2.7	$1.2 x10^{-64}$	99
	YWHAG	tyrosine 3-monooxygenase/tryptophan 5-monooxygenase activation protein gamma	4.6	$1.5x10^{-168}$	1	2.2	$1.4 x10^{-64}$	101
	HIF1A	hypoxia inducible factor 1 alpha subunit	2.2	$1.1x10^{-51}$	82	2.3	$3.4 x10^{-51}$	149
	MARCKS	myristoylated alanine rich protein kinase C substrate	1.3	$2.4 x10^{-06}$	932	2.9	$5.2 x10^{-84}$	40
	NSUN2	NOP2/Sun RNA methyltransferase family member 2	2.1	$1.1 x10^{-63}$	51	1.4	$4.1 x10^{-11}$	1067
	HNRNPD	heterogeneous nuclear ribonucleoprotein D	2.2	$3.0 x10^{-64}$	48	1.4	$8.3 x10^{-11}$	1089
	DEK	DEK proto-oncogene	1.3	$1.8 x10^{-06}$	916	2.8	$1.4 x10^{-73}$	61
	YWHAB	tyrosine 3-monooxygenase/tryptophan 5-monooxygenase activation protein beta	2.1	$7.4 x10^{-67}$	40	1.5	$8.6 x10^{-18}$	772
	ANP32E	acidic nuclear phosphoprotein 32 family member E	2.4	$5.0 x10^{-62}$	56	1.6	$5.2 x10^{-20}$	669
	CALM1	calmodulin 1	1.3	$2.4 x10^{-7}$	846	3.3	$2.7 x10^{-139}$	6
	PCNA	proliferating cell nuclear antigen	2.3	$7.9 x10^{-64}$	50	1.3	$8.4 x10^{-08}$	1292
	HMGB2	high mobility group box 2	1.3	$5.8 x10^{-07}$	878	5.3	$2.8 x10^{-162}$	3
phophorylation upregulated in docetaxel-resistant PC3 (See Table S2)	PKP4	plakophilin 4	1.5	$8.1 x10^{-13}$	538	-	-	-
	AXL	AXL receptor tyrosine kinase	2.3	$5.9 x10^{-30}$	198	-	-	-
	HIPK3	homeodomain interacting protein kinase 3	2.3	$3.9 x10^{-28}$	219	-	-	-
	PTPRA	protein tyrosine phosphatase, receptor type A	1.7	$5.9 x10^{-25}$	271	-	-	-
	RPLP0	ribosomal protein lateral stalk subunit P0	-	-	-	1.4	$5.6 x10^{-10}$	1129
	VIM	vimentin	-	-	-	1.7	$2.1 x10^{-30}$	361
	PTK2	protein tyrosine kinase 2	-	-	-	1.3	$1.3 x10^{-08}$	1226
	ATP1A1	ATPase Na+/K+ transporting subunit alpha 1	-	-	-	1.3	$1.6 x10^{-09}$	1157
	DCBLD2	discoidin, CUB and LCCL domain containing 2	-	-	-	1.8	$7.9 x10^{-26}$	470
	ANTXR2	anthrax toxin receptor 2	-	-	-	1.8	$1.3 x10^{-32}$	326
	LPXN	leupaxin	-	-	-	1.5	$2.1 x10^{-08}$	1249
	ADAM9	ADAM metallopeptidase domain 9	1.5	$2.9 x10^{-18}$	400	1.3	$1.8 x10^{-08}$	1243
	SIRPA	signal regulatory protein alpha	1.4	$1.8 x10^{-09}$	689	1.4	$6.5 x10^{-13}$	971
	SET	SET nuclear proto-oncogene	1.6	$8.3 x10^{-23}$	313	1.4	$2.9 x10^{-12}$	1005
protein upregulated in docetaxel-resistant PC3 (see Table S2)	STX2	syntaxin 2	1.7	$3.2 x10^{-22}$	319	2.6	$1.0 x10^{-73}$	60
	GOLIM4	golgi integral membrane protein 4	1.6	$1.5 x10^{-16}$	432	1.8	$2.7 x10^{-24}$	515
	SACS	sacsin molecular chaperone	1.5	$1.7 x10^{-03}$	1282	4.3	$1.6 x10^{-33}$	312
	PICALM	phosphatidylinositol binding clathrin assembly protein	1.4	$4.3 x10^{-13}$	529	1.5	$9.8 x10^{-15}$	887
	TMEM65	transmembrane protein 65	2.8	$4.3 x10^{-52}$	81	1.9	$1.8 x10^{-25}$	484
	ANXA6	annexin A6	1.5	$3.9 x10^{-18}$	403	1.7	$9.7 x10^{-24}$	535
within top 100	EDIL3	EGF like repeats and discoidin domains 3	2.9	$1.3 x10^{-81}$	25	3.9	$1.4 x10^{-106}$	21

downregulated mRNA	COL6A3	collagen type VI alpha 3 chain	3.8	3.6 x10^{-52}	80	6.9	1.6 x10^{-177}	2
	FSTL1	follistatin like 1	3.1	5.2 x10^{-136}	7	2.6	3.9 x10^{-107}	20
	ATP11C	ATPase phospholipid transporting 11C	3.3	2.2 x10^{-70}	34	3.2	2.5 x10^{-69}	81
	TOMM20	translocase of outer mitochondrial membrane 20	2.2	1.0 x10^{-59}	60	2.6	7.6 x10^{-82}	43
	GHITM	growth hormone inducible transmembrane protein	3.1	1.3 x10^{-122}	11	2.6	1.4 x10^{-64}	100
	NEGR1	neuronal growth regulator 1	4.1	7.8 x10^{-109}	14	4.2	3.1 x10^{-122}	13
	MBOAT2	membrane bound O-acyltransferase domain containing 2	2.4	1.0 x10^{-66}	41	3.5	2.9 x10^{-131}	9
	SEC23A	Sec23 homolog A, coat complex II component	2.3	7.0 x10^{-76}	31	2.8	1.2 x10^{-99}	23
	MRC2	mannose receptor C type 2	3.5	2.4 x10^{-76}	30	3.7	5.8 x10^{-70}	79
>2 fold difference	SULF2	sulfatase 2	4.2	7.6 x10^{-161}	5	2.2	6.6 x10^{-51}	150
	FBN2	fibrillin 2	2.8	5.0 x10^{-42}	120	4.3	1.0 x10^{-64}	97
	CERK	ceramide kinase	2.0	4.3 x10^{-30}	196	3.1	8.6 x10^{-87}	39

Table S2.
List of proteins with significantly upregulated tyrosine phosphorylation or protein levels in docetaxel-resistant PC3 (referred to as PC3Rx) compared to PC3. Data was obtained by quantitative mass spectrometry-based phospho-proteomic profiling of these cell lines (described in Lee *at al* 2014 Mol Cancer Ther)[1].

Findings from phospho-proteomic profiling	Protein	Phospho-tyrosine position	Fold difference of SILAC* ratios (PC3Rx/PC3)
Tyrosine phosphorylation levels upregulated in PC3Rx compared to PC3 ($p<0.05$)	PKP4	Y478	1.25
	AXL	Y702	2.03
	HIPK3	Y359	1.95
	PTPRA	Y798	2.73
	RPLP0	Y24	1.34
	VIM	Y61	2.22
	PTK2	Y397, Y577, Y576	1.65, 1.51, 1.42
	ATP1A1	Y260	1.97
	DCBLD2	Y764	1.41
	ANTXR2	Y380	1.81
	LPXN	Y27	3.86
	ADAM9	Y815	1.72
	SIRPA	Y496	1.83
	SET	Y133	3.00
Protein levels upregulated in PC3Rx compared to PC3 ($p<0.05$)	STX2		2.69
	GOLIM4		2.47
	SACS		3.50
	PICALM		2.24
	TMEM65		2.34
	ANXA6		2.16
	VIM		2.26
	LPXN		3.30
	ANTXR2		2.18

*SILAC, stable isotope labeling with amino acids in cell culture

[1]Lee BY, Hochgrafe F, Lin HM, Castillo L, Wu J, Raftery MJ, Martin Shreeve S, Horvath LG, Daly RJ. Phosphoproteomic profiling identifies focal adhesion kinase as a mediator of docetaxel resistance in castrate-resistant prostate cancer. *Mol Cancer Ther* 2014;13(1):190-201
